# Serving Children and Adolescents in Need during the COVID-19 Pandemic: Evaluation of Service-Learning Subjects with and without Face-to-Face Interaction

**DOI:** 10.3390/ijerph18042114

**Published:** 2021-02-22

**Authors:** Li Lin, Daniel T. L. Shek

**Affiliations:** Department of Applied Social Sciences, The Hong Kong Polytechnic University, Hong Kong, China; daniel.shek@polyu.edu.hk

**Keywords:** service learning, online teaching and learning, higher education, COVID-19 pandemic, positive youth development

## Abstract

The coronavirus disease (COVID-19) outbreak has posed a great challenge to teaching and learning activities in higher education, particularly for service-learning subjects that involve intensive human interaction. Although service-learning may be transformed to a virtual mode in response to the pandemic, little is known about the impact of this new mode on student learning and well-being. This paper reports a university credit-bearing service-learning subject that involves services toward needy children and adolescents in a non-face-to-face mode under COVID-19 pandemic. We examined the effectiveness of this subject by comparing it with the same subject delivered via a face-to-face mode. Objective outcome evaluation via a pretest-posttest comparison (N = 216) showed that the students who took service-learning subjects with and without face-to-face interaction showed similar positive changes in positive youth development competences, service leadership qualities, and life satisfaction. Subjective outcome evaluation (N = 345) also showed that most students were satisfied with the subject, instructors and benefits regardless of the service mode. The findings highlight the important role of non-face-to-face service learning in promoting college students’ positive growth and well-being.

## 1. Introduction

Due to the coronavirus disease (COVID-19) pandemic, educational institutions worldwide, from kindergartens to universities, have been forced to move their classes to virtual platforms [1,2,3]. In an effort to contain the spread of COVID-19 via social contact, distance teaching or e-learning, including synchronous online classes and asynchronous lecture videos, is often adopted to replace traditional face-to-face (FTF) teaching in a physical classroom [4,5]. The sudden transition to distance teaching raises concerns about its educational quality and its impact on students’ well-being [6,7]. Thus, it is critical to provide evidence on how these new teaching approaches influence students’ learning and well-being.

Compared with the FTF teaching/learning mode, a lack of FTF interaction poses a greater challenge to the delivery of service learning, which involves experiential learning through engagement in community service [8]. Although many evaluation studies have investigated FTF service learning [9,10,11], whether service learning can be successfully implemented without FTF interaction is unknown. This paper reports how a credit-bearing service-learning subject was transformed from an FTF mode into a non-FTF (nFTF) mode in response to the COVID-19 pandemic. Despite a few case studies investigating virtual service learning during the pandemic [12,13,14], little is known about whether nFTF service learning yields positive learning outcomes and brings benefits or costs on students’ well-being. In this study, we used objective outcome evaluation to examine whether the nFTF service-learning subject demonstrated positive changes in student learning and life satisfaction. Besides, we also employed subjective outcome evaluation to examine whether nFTF service-learning subject was well received by the students. We also compared nFTF service learning with the traditional FTF mode in terms of different evaluation findings. The current study will provide educators and practitioners with insights regarding the transformation of the service-learning pedagogy and promotion of young people’s well-being in higher education.

### 1.1. Definition and Benefits of Service Learning

Service learning is a pedagogy that integrates community service with academic learning [9,15]. This pedagogy is grounded in Dewey’s experiential learning theory [16], which advocates “learning by doing” and Kolb’s experiential learning cycle [17]. According to Kolb [17], students who participate in service learning will go through a cycle of four stages. They first create experiences by applying their academic concepts into serving the community, and then, they observe and reflect on these experiences. Such reflective observation will lead to a transformation in their understanding of the concepts, which can be used in future situations that result in new experiences. One key component of service learning is mutual benefit or reciprocity [18]. Service learning is differentiated from volunteering or community service, which often does not include structured educational components [19]. Under service learning, students are expected to fulfill both the identified community needs and educational goals. Therefore, both service recipients and service providers will benefit from the activities [9,20].

Previous studies have revealed that service learning relates to a variety of beneficial outcomes in students’ intellectual development, personal development, civic development, and other social attitudes [9,10,21,22]. For example, Salam’s systematic review [23] identified multiple potential benefits of service learning in students, including cognitive development, communication and interpersonal skills, social and civic responsibility, leadership, and academic learning. Celio’s meta-analysis [9] of 62 studies showed that service learning had small positive effects on attitudes about self, attitudes toward school and learning, civic engagement, social skills, and academic performance. Yorio’s meta-analysis [22] of 40 studies also showed that engagement in service learning had small positive effects on students’ understanding of social issues (e.g., cultural awareness and sensitivity, understanding of community needs), personal insights (e.g., awareness of personal strengths and weakness, self-esteem), and cognitive development (e.g., writing skills, problem-solving skills, academic performance). Additionally, theoretically speaking, service learning is a vehicle for positive youth development which underscores the importance of youth competencies and potentials [24,25]. There are research findings showing that possession of positive youth development competencies contributes to the well-being of adolescents [26,27]. Therefore, engagement in service learning possibly contributes to students’ well-being.

### 1.2. Alternative Mode of Service Learning in Response to COVID-19 Pandemic

The benefits that have been reported in the existing studies are largely based on traditional service-learning activities that involve FTF interactions among students, teachers, service partners, and service recipients. When the COVID-19 pandemic rendered the suspension of FTF teaching and implementation of mandatory social distancing measures, the benefits of service learning become questionable. Rather than simply canceling service-learning activities, educators have transformed direct services, in which students conduct on-site services, into indirect or remote services [14]. For example, DePaul University provided multiple indirect service opportunities, such as face-mask making (for elderly and health professionals), letter writing (for health professionals), research help, video making, and small donations [14]. Johns Hopkins School of Nursing formed a partnership with Baltimore Neighbors Network to provide their nursing students with an opportunity to serve and learn by offering phone-based support to the elderly in Baltimore during the pandemic [28].

Before the COVID-19 pandemic, some scholars had been aware of the advantages of incorporating the online component into service learning [29,30,31], such as engaging a wider range of the population, including students who receive distance education and service targets who have difficulties visiting service sites. Based on a literature review, Waldner [31] identified four ways of online service learning, including (1) learning online and serving offline, (2) learning offline and serving online, (3) learning and serving partially online and partially offline, and (4) both learning and serving online. However, these virtual types of service learning remain rare in higher education, possibly due to the challenges in curriculum design, teacher training, technical support, and collaboration with service partners.

Although nFTF service learning appears feasible, evidence of its effectiveness in enhancing student learning is scarce. An evaluation study is a “systematic investigation to determine the success of a specific program” [32] (p.149). It usually serves two objectives: to obtain information that helps improve the program (i.e., formative evaluation) and to show the outcomes and impacts of the program (i.e., summative evaluation; [33]). A handful of evaluation studies on nFTF service-learning programs have mainly used interviews [12,13] and student reports to collect students’ perceptions and attitudes about the service and its benefits [29]. These studies have provided preliminary evidence for the effectiveness of nFTF service learning in promoting student learning, but the results have been based on students’ subjective perceptions only. Furthermore, without comparisons with traditional service learning, it is unclear which mode is more advantageous [31], but such comparative studies are almost nonexistent. The only exception, which compared the students’ perceptions of benefits (i.e., gains in practical skills, interpersonal skills, citizenship, and personal responsibility) between traditional mode and online mode, found similar perceived benefits across the two service modes [34]. However, similar to other studies, this study only investigated students’ perceived benefits after the course through survey. In this study, we argue that it is crucial to conduct evaluation studies that validate the effectiveness of nFTF service learning by using different approaches beyond student perception. In addition, no study has been reported in different Chinese societies, which comprise roughly one-fifth of the world’s young people.

### 1.3. Current Study

#### 1.3.1. Service-Learning Subject under Study

In light of the gaps mentioned above, we examined the effectiveness of an nFTF service-learning subject in this study. The service-learning subject under examination was a credit-bearing subject titled “Service Leadership Through Serving Children and Families with Special Needs” which was based on the Service Leadership Theory [35,36]. Due to the transformation of the economic structure from manufacturing economies to service economies, Chung and Bell [35] proposed the concept of “service leadership” in which a leader prioritizes service over personal interests. In this new conceptualization, leadership “is about satisfying needs by consistently providing quality personal service including one’s self, others, groups, communities, systems, and environments” [36,37]. Central to this leadership model are three determinants of effective service leadership: moral character, caring disposition, and leadership competences. Effective leaders in service economies are expected to be competent, caring and acting morally. In addition, the service leadership theory adopts a broad definition of leadership by highlighting that everyone can be a leader regardless of his/her position and that leading oneself (i.e., self-leadership) is an important part of effective leadership [38]. Students who take this subject are expected to acquire knowledge, attitudes, and skills of service leadership; apply them in their community service; and ultimately improve their positive youth development competences and service leadership qualities. The details of this subject’s learning outcomes can be seen in the online Supplementary Material (see Table S1).

The teaching and learning period for this service-learning subject usually lasts for two semesters, from January to July. First, students attend three lectures to learn basic knowledge about service leadership. Next, they attend four workshops on the needs of service targets, skills to facilitate service activities, and skills for service planning. The service targets are children and adolescents with various special needs who live in Hong Kong and mainland China. In Hong Kong, the service targets include school children and adolescents with emotional or behavioral problems from Society of Boy’s Centres, pre-school children with intellectual difficulties or development disorders from Heep Hong Society, and adolescents from local schools who are underachieving academically with social deprivation [11,20]. Service activities are designed according to the specific needs of the service targets, such as tutorial, extracurricular class, day camp, and campus visit. In mainland China, service targets include migrant children who moved from a village to the city with their parents or left-behind children whose parents moved to the city to make a better living. These children are regarded as vulnerable, given their lack of social capital and educational resources [39,40]. Students organize a five-day summer camp, in which they offer multiple programs in English, science, health, and personal development and also train children to perform in a closing ceremony. Several preliminary evaluation studies have tested the effectiveness of the subject in the location of Hong Kong [11,20,41]. The results showed that students experienced significant improvement in cognitive development, positive youth development competence, and service leadership qualities but not in civic development. Additionally, most students reported positive perceptions of the teaching quality and benefits gained from the subjects. Additionally, students in earlier cohorts also showed positive perceptions toward the subject in Hong Kong service site [42].

Due to the COVID-19 pandemic in 2020, all lectures and workshops were conducted via a real-time web conferencing tool (Blackboard Collaborate Ultra) which allows teachers and students to communicate synchronously. Additionally, service activities were changed to indirect services. There are three service sites under this subject. In Hong Kong, the service activities include making tutorial videos (e.g., video of career and life planning) and holding online learning workshops (e.g., English learning workshop). In mainland China, the summer camp was moved to VooV Meeting, a video conferencing platform powered by Tencent. Children from Xi’an were grouped into several classrooms and attended the online lessons together with one class teacher present via classroom devices. Children from Chengdu attended the online lessons at home using their personal devices, such as a laptop, tablet, or smartphone. Obviously, it is important to know whether such a transformation would influence the learning benefits of the service-learning subject and whether it would bring benefits or costs on students’ well-being.

#### 1.3.2. Research Questions and Hypotheses

To triangulate the findings, two evaluation approaches were used to examine the effectiveness of nFTF service learning amid the pandemic: objective outcome evaluation (OOE) and subjective outcome evaluation (SOE). OOE concerns the change in student attributes before and after participating in service learning [43,44]. This study used a one-group pretest-posttest design. As the subject’s learning outcomes mainly include positive youth development competence and service leadership qualities, we investigated the changes in these learning outcomes for students. In addition, we measured students’ life satisfaction as an index of their overall well-being [45]. Rooted in the customer satisfaction approach, SOE is concerned with whether the participants feel contented with the program and whether their needs have been satisfied [46]. This approach is widely used in education for evaluating teaching quality [47] as well as in human services [48]. This study used a structured questionnaire to solicit students’ feedback on the quality of the service-learning subject. Overall, this study sought to answer four research questions:

*Research Question 1*: Do students show positive changes after taking the FTF and nFTF service-learning subjects?

**Hypothesis** **1**. *In accordance with previous studies [11,41], we proposed that students taking the FTF and nFTF service-learning subjects would show positive pretest-posttest changes (Hypothesis 1, respectively)*.

*Research Question 2*: What are the perceptions of the students taking FTF and nFTF service-learning subjects regarding the subject, teachers, and benefits?

**Hypothesis** **2**. *Consistent with previous findings [11,42], we expected that the majority of the students in both FTF and nFTF service-learning subjects would have positive perceptions about the quality of both FTF (Hypothesis 2) and nFTF (Hypothesis 2) service-learning subjects. Besides, while there are theories and findings to support the effectiveness of FTF service learning [9,21,22], there are also arguments and evidence highlighting the value of nFTF service learning [28,31]. As there are both pros and cons for FTF and nFTF service learning, we examined the following research questions with competing hypotheses*.

*Research Question 3*: Are students taking FTF service learning and nFTF service learning subjects different in the objective outcomes?

**Hypothesis** **3**. *Students taking part in FTF service-learning subject would show greater (vs. smaller) positive changes than those taking nFTF service-learning subject*.

*Research Question 4*: Are students taking FTF service learning and nFTF service learning different in the subjective outcomes?

**Hypothesis** **4**. *Students taking FTF service learning would report better (vs. worse) perceptions of the subject qualities than those taking nFTF service learning*.

## 2. Materials and Methods

### 2.1. Participants and Procedure

Students who took the service-learning subject were invited to participate in both OOE (i.e., pretest-posttest evaluation) and SOE (i.e., post-course evaluation). For OOE, a total of 282 students completed the pretest, and 316 students completed the post-test (see Table 1). Successful matching was made for 216 cases (mean age = 19.78 ± 1.61 years; female = 122), in which 86 students (mean age = 19.92 ± 1.55 years; female = 45) took the traditional service-learning subject with FTF interaction, while 130 students (mean age = 19.68 ± 1.65 years; female = 77) took the service-learning subject without FTF interaction. The age (*t*(212) = 1.05, *p* = 0.30) and gender composition (*χ*^2^_(1)_ = 1.00, *p* = 0.32) of participants in the FTF and nFTF modes were comparable. For the SOE, 345 students completed the questionnaires, and 125 of them took the FTF service-learning subject, 220 took the nFTF service-learning subject. To protect the privacy of the students in the course evaluation, no demographic information was collected for the SOE.

All the evaluation questionnaires were posted on an online teaching and learning platform (i.e., Blackboard), and students completed the questionnaires in a self-administrative manner, voluntarily without incentive. For the OOE, the pretest was conducted within the first three weeks of the teaching period before any service was conducted, and the posttest was conducted after all the service and teaching activities had been completed. For SOE, students filled out the questionnaires after all the service activities were completed. Informed consent was obtained from all participants involved in the study before the distribution of these two evaluation questionnaires.

### 2.2. Measures

To examine the pretest-posttest changes, participants completed an OOE form. Additionally, participants reported their evaluation of the program qualities by completing an SOE form after concluding the subject. All forms were presented in both English and Chinese and have been repeatedly used with good psychometric properties to measure the course effectiveness of leadership subjects with and without service-learning components [20,49,50].

#### 2.2.1. Objective Outcome Evaluation (OOE)

Participants completed an identical OOE form before and after the service activities. This evaluation form assessed participants’ positive youth development competencies, service leadership qualities, and life satisfaction on a 6-point scale (1 = strongly disagree; 6 = strongly agree). First, positive youth development attributes were measured by 36 items adapted from the Chinese Positive Youth Development Scale [51]. Two or three items each were used to measure participants’ social competence, emotional competence, cognitive competence, behavioral competence, moral competence, self-determination, clear and positive identity, belief in the future, spirituality, and resilience [11]. Mean scores were taken to represent each positive youth development attribute, and a mean score was taken to indicate the overall level of positive youth development. The overall scale and the majority of the subscales yielded satisfactory internal consistencies for both pretest and posttest evaluations (see Table 2). Second, the service leadership scale was used to measure students’ self-leadership, caring disposition, moral character, and service leadership beliefs. Mean scores were taken to represent each aspect of service leadership qualities, and a mean score was taken to indicate the overall service leadership. The scale and subscales all demonstrated good internal consistencies (see Table 2) at both pretest and posttest. Finally, the five-item Satisfaction with Life Scale was used to measure students’ overall evaluation of their life conditions [52]. This scale was translated into Chinese by Shek [53] with good psychometric properties. All the items were averaged to indicate individuals’ life satisfaction level. This scale demonstrated good internal consistency in the current study. Sample items from the OOE form are presented in the online Supplementary Material (see Table S2).

#### 2.2.2. Subjective Outcome Evaluation (SOE)

An SOE form was used for students to report their evaluation of the subject. This form comprised one structured questionnaire concerning students’ perceptions of the subject content (10 items, e.g., curriculum objectives, curriculum design, and class activities), the teacher’s performance (10 items, e.g., mastery of curriculum, teaching skills, and care for students), and the effectiveness of the subject in enhancing positive youth development competencies and service leadership qualities (18 items, e.g., social competence, critical thinking, leadership competences). Students were asked to rate each statement about the course on a 5-point scale (1 = strongly disagree; 5 = strongly agree). Mean scores were taken to represent students’ evaluation of the three aspects of the subject. Additionally, participants reported, through the three items, their overall satisfaction with the course (1 = Very dissatisfied; 5 = Very satisfied), willingness to recommend a friend take this course (1 = Definitely will not, 5 = Definitely will), and willingness to participate in a similar course (1 = Definitely will not, 5 = Definitely will). Previous studies have shown good psychological properties for this scale [41]. The reliabilities of subscales in the current study were all good (see Table 3).

## 3. Results

### 3.1. Objective Outcome Evaluation

Before testing the pretest-posttest changes, we examined whether there were any differences in students’ developmental outcomes at the pretest (i.e., baseline) according to service mode (nFTF vs. FTF). A multivariate analysis of variance (MANOVA) was conducted with 10 positive youth development attributes as dependent variables and service mode as a fixed factor. Overall, the results showed no significant differences in service mode for positive youth development attributes (*F*_(10,269)_ = 1.28, *η*_p_^2^ = 0.05; *p* > 0.05). Similarly, MANOVA on four service leadership qualities (i.e., self-leadership, moral character, caring disposition, and service leadership beliefs) did not detect significant difference (*F*_(4274)_ = 2.07, *η*_p_^2^ = 0.03; *p* > 0.05). Lastly, a univariate analysis of variance (ANOVA) on life satisfaction did not reveal a significant difference in service mode (*F*_(1279)_ = 0.86, *η*_p_^2^ = 0.003; *p* > 0.05). These findings suggest that the baselines of student qualities were similar in service-learning subjects adopting the nFTF and FTF modes.

Next, a series of two-way repeated measure ANOVAs were conducted to examine pretest-posttest changes in the developmental outcomes and differences between students taking FTF and nFTF subjects. Each analysis targeted one attribute. The results are presented in Table 4. We found significant positive changes in the overall levels of positive youth development competencies, service leadership qualities, and life satisfaction from the pretest to posttest. Positive changes were observed in all the individual indicators of positive youth development competencies, all indicators of service leadership qualities. However, there were no interaction effects between change and service mode (FTF vs. nFTF) in all variables except clear and positive identity. Students taking nFTF subject showed greater positive change in clear and positive identity than students taking FTF subject. The positive changes in other variables were similar in the students taking FTF and those taking nFTF subject.

To summarize, the findings of OOE supported our hypotheses that students who took service-learning subjects experienced positive changes in positive youth development competences, service leadership qualities, and well-being (Hypotheses 1a and 1b), but did not support the hypotheses about the difference between the two service modes (Hypotheses 3a and 3b).

### 3.2. Subjective Outcome Evaluation

Table 3 presents the mean scores of subjective outcome evaluation and Table 5 presents the frequency of students’ positive response to each item. These mean scores revealed that students had very positive perceptions on the service-learning subjects. Additionally, over four fifth of the students reported positive evaluation toward subject content, teachers’ performance, and subject effectiveness. For example, 84.6% and 90.3% of the students in FTF subject and nFTF subject agreed that the design of the curriculum was very good. A total of 92.8% and 94.5% of the students in FTF subject and nFTF subject, respectively, considered that the teaching skills of the lecturers were good. A total of 92.0% and 93.2% of the students in FTF subject and nFTF subject, respectively, perceived that their social competence has been enhanced. Therefore, we found evidence to support Hypotheses 2a and 2b.

Furthermore, to explore if students’ subjective outcome evaluation varied across FTF vs. nFTF service-learning subjects, we first performed three sets of ANOVA on the perceptions of subject content, teacher’s performance, and subject effectiveness, respectively. The service mode did not make any difference in the students’ evaluations of subject content (*F*_(3343)_ = 2.41, *p* > 0.05, *η*_p_^2^ = 0.01), teacher’s performance (*F*_(3343)_ = 0.02, *p* > 0.05, *η*_p_^2^ = 0.00), and subject effectiveness (*F*_(3343)_ = 1.72, *p* > 0.05, *η*_p_^2^ = 0.01). Second, we conducted three sets of ANOVA on overall satisfaction, willingness to recommend this subject, and willingness to take a similar subject, respectively. The results showed students who used the nFTF mode were more satisfied with the subject mode (*F*_(1342)_ = 3.87, *p* < 0.05, *η*_p_^2^ = 0.01) and were more willing to recommend this subject to their friends than those who used the FTF mode (*F*_(1342)_ = 8.18, *p* < 0.01, *η*_p_^2^ = 0.02). However, there was no difference in service mode for the willingness to take a similar subject.

Altogether, these findings support our hypotheses that students who use both nFTF and FTF modes for service learning reported positive evaluations of the subject qualities (Hypotheses 2). However, Hypothesis 4 that nFTF subject was better than FTF subject was only partially supported.

## 4. Discussion

Distance learning, e-learning, and blended learning that mixes live classroom with e-learning components have been increasingly popular in higher education due to the rapid advancement of information and communication technologies [54,55]. However, service learning as an experiential learning pedagogy, aimed to promote students’ positive development and needy people’s quality of life, seems to be left behind in the innovation of distance learning and e-learning. The current study provides evidence of the effectiveness of adopting these new ways of teaching and learning in the context of service learning.

In this study, we found that students who took the FTF service learning and nFTF service learning both showed positive changes in positive youth development competencies, such as emotional competence and resilience; service leadership qualities, such as self-leadership and caring disposition; and life satisfaction. However, except clear and positive identity, no significant differences were found across the two service modes. The findings suggest that transforming service learning to an nFTF mode may not impede student learning and well-being. In the current case, despite no FTF interaction with teachers and service recipients, students were allowed to meet teachers through online platforms and provide indirect services. They were still able to achieve the intended learning outcomes of the subject in terms of knowledge acquisition and application of academic knowledge in community service. Additionally, compared to the traditional mode of service learning, they encountered many new problems due to the ever-changing situation of the pandemic and technical barriers. Nevertheless, these problems could be turned into opportunities to learn. When working with their group mates to solve these problems, the students can achieve a major intended learning outcome—improvement of service leadership capacities (i.e., caring, moral character, and multiple competences). Furthermore, when this painstaking process is translated into a gain in competencies and capacities, students probably feel good about their lives. The meaningful engagement in community, especially under a seemingly “impossible” situation, probably brings students a sense of enjoyment and achievement. Altogether, it improves their well-being. Numerous studies have reported that college students across the globe have suffered from heightened psychological distress and dampened well-being due to the prevalence of infectious disease, school closures, and difficulty in adjusting to new learning modes during the pandemic [6,7]. Therefore, the positive changes observed in the students of this service learning were inspiring. Service learning could help young people improve their competencies and leadership and enhance their well-being even during the pandemic. These findings suggest that engagement in service learning might be a protective factor that counteracts the harmful impacts of disease outbreak on young people’s well-being. We encourage future studies to examine how service learning helps young people reap benefits of positive development under COVID-19 pandemic conditions.

Additionally, we found that students reported similar positive evaluations of the two modes of service learning. Most of them considered the subject content and teacher’s performance to be good. Additionally, they considered the subject beneficial for their improvement in multiple competencies and leadership capacities. More surprisingly, students taking nFTF subjects reported even higher overall satisfaction and willingness to recommend this subject to their friends than students taking FTF subject. In contrast with reports indicating university students’ worry, distress, and discontent with e-learning [56,57], this study showed high levels of satisfaction among students. This nFTF mode of service learning incorporated helpful practices that facilitated online learning, such as adjusting teaching content and activities for an effective delivery of teaching information, providing adequate support from teacher and teaching assistants to students, and preparing contingency plans to deal with unexpected incidents due to technical problems [58]. Also, this subject emphasized reflection, which has been found to enhance the effectiveness of service learning [22,30]. Students took part in a group reflection to discuss their problems and gains after each service activity, and they wrote individual reflective journals to report their service process and insights. Admittedly, many factors can affect the success of distance education and e-learning [59]. Future studies can explore what contributes to the high effectiveness of an nFTF service-learning program.

### 4.1. Theoretical and Practical Implications

This study advances the literature by comparing nFTF service learning and FTF service learning. E-learning or distance learning is booming in higher education, particularly under COVID-19. Economic analysis has found that use of online learning may reduce the cost of higher education, which brings hope to students with economic difficulty [60]. However, research that compares their effectiveness with that of traditional classes is rare, which hinders the extension of theory about higher education into virtual context. Also, findings from these studies have been mixed. Some studies have revealed a better learning outcome for in-class learning [61], some have revealed a better outcome for e-learning [62], while some have not shown any significant difference between the two [63,64]. Consistent with previous research in the learning of clinical skills [65], this pioneering study shows similar effectiveness between nFTF service learning and FTF service learning. This study suggests that nFTF mode of teaching and learning could be included into the theorizing and modeling of quality higher education, especially for nurturing life skills or soft skills from the positive youth development perspective [27,66]. In the case of service learning, educational scholars need to further test whether previous theoretical perspectives (e.g., reciprocity of service learning [18]) apply to nFTF service and refine the theory, particularly with reference to the theoretical mechanisms involved.

Practically, these findings suggest that nFTF may be an alternative mode of service learning that can be used in the future, even after the pandemic. It allows students to serve people living in remote villages without traveling as long as they can access the Internet. It also allows students to serve people with disabilities who have difficulty in commuting from their home to the service site. Admittedly, nFTF service learning evokes new challenges in a variety of issues, such as low effectiveness of communication and unsatisfactory online curriculum [67], and it requires professional teacher training. Nevertheless, the current findings may empower educators and practitioners to reimagine the service-learning pedagogy and extend the ways they engage the community and students in the service.

### 4.2. Limitations and Future Directions

The current findings should be interpreted with caution. First, we could not draw a causal conclusion about the effect of service learning on students’ changes in learning outcomes because the current study was not based on an experimental design. Randomized assignment of student participants is not applicable to testing the effectiveness of a credit-bearing course, as registration in the course depends on students’ preferences and schedules. Also, as student participation in this study was voluntary, it was difficult to recruit two comparable samples that shared similar demographic and academic backgrounds in the current situation. Future studies will benefit from a quasi-experiment that can match the background of students who take this subject with a sample who do not take a service-learning subject but share comparable background such as similar gender composition, year of study, and faculty (see an example [45]). Second, students’ motivation to complete the survey may influence the results, but this was not investigated in this study. Because completion of the survey was voluntary, students who took this subject more seriously may have been more likely to help with the survey. Future studies could link students’ grades in the subject to these evaluation data and validate if only “high performing” students experience positive changes and have positive perceptions. Finally, this study did not collect feedback from service recipients and service partners. Concerns still remain that indirect service, though bringing learning benefits to students, may not sufficiently fulfill community needs [12]. In our current case, it is unknown whether those needy children and adolescents obtained more gains from FTF activities than from nFTF activities. As reciprocity is an essential feature of service learning [18], more evaluation approaches are needed to solicit evidence from the perspectives of service recipients and service partners [20].

## 5. Conclusions

Despite these limitations, this study serves as a timely response to the call for understanding young people’s learning and well-being under the COVID-19 pandemic [68]. More importantly, we found that, similar to traditional service learning, students taking part in service learning without FTF interactions experienced positive growth and showed favorable evaluations. These findings indicate the feasibility and effectiveness of adopting distance learning and indirect service in service learning. Accordingly, scholars and educators may further think about how to innovate the service-learning pedagogy to benefit a wider range of students and communities.

## Figures and Tables

**Table 1:** Descriptive information of OOE and SOE.

Academic Year	Location of Service Targets	Service Mode	Pretest	Posttest	Matched Cases of OOE	Valid Cases of SOE	Enrollment Number
2017/18	Hong Kong	FTF	48	36	26	46	82
2017/18	Mainland China (Xi’an)	FTF	24	42	24	43	43
2018/19	Hong Kong	FTF	59	40	36	36	70
2019/20	Hong Kong	nFTF	66	77	45	100	119
2019/20	Mainland China (Chengdu)	nFTF	55	82	55	82	82
2019/20	Mainland China (Xi’an)	nFTF	30	39	30	38	41

Note: OOE = objective outcome evaluation; SOE = subjective outcome evaluation; FTF = face-to-face; nFTF = non-face-to-face.

**Table 2:** Descriptive information of the outcome variables on objective outcome evaluation variables.

Dependent Variables	Pre-Test				Post-Test			
	Mean (SD)			α	Mean (SD)			α
	Overall	FTF	nFTF	Overall	Overall	FTF	nFTF	Overall
PYD (overall level)	4.65 (0.53)	4.59 (0.50)	4.71 (0.56)	0.93	4.94 (0.54)	4.84 (0.57)	5.01 (0.51)	0.93
Social competence	4.87 (0.67)	4.82 (0.67)	4.92 (0.67)	0.87	5.15 (0.64)	5.01 (0.67)	5.24 (0.62)	0.91
Emotional competence	4.66 (0.61)	4.60 (0.61)	4.71 (0.61)	0.67	4.98 (0.69)	4.83 (0.69)	5.06 (0.67)	0.82
Cognitive competence	4.76 (0.62)	4.70 (0.56)	4.82 (0.67)	0.80	5.09 (0.61)	4.95 (0.64)	5.17 (0.58)	0.86
Behavioral competence	4.71 (0.70)	4.65 (0.68)	4.76 (0.71)	0.62	5.06 (0.67)	4.92 (0.67)	5.15 (0.66)	0.73
Moral competence	4.75 (0.62)	4.64 (0.59)	4.84 (0.63)	0.54	4.91 (0.64)	4.90 (0.66)	4.92 (0.62)	0.41
Self-determination	4.60 (0.72)	4.50 (0.73)	4.68 (0.71)	0.77	4.96 (0.67)	4.82 (0.74)	5.03 (0.61)	0.77
Clear and positive identity	4.23 (0.92)	4.18 (0.85)	4.27 (0.98)	0.77	4.67 (0.88)	4.52 (0.92)	4.77 (0.84)	0.85
Belief in the future	4.92 (0.68)	4.85 (0.63)	4.98 (0.72)	0.77	5.16 (0.63)	5.09 (0.66)	5.20 (0.61)	0.75
Spirituality	4.37 (0.80)	4.37 (0.81)	4.37 (0.79)	0.58	4.58 (0.79)	4.54 (0.88)	4.60 (0.73)	0.57
Resilience	4.69 (0.74)	4.59 (0.73)	4.78 (0.75)	0.81	4.91 (0.72)	4.83 (0.78)	4.95 (0.68)	0.83
Well-being								
Life satisfaction	4.00 (0.94)	3.94 (0.97)	4.05 (0.91)	0.87	4.42 (0.94)	4.35 (1.01)	4.46 (0.90)	0.88
Service leadership (overall level)	4.77 (0.52)	4.68 (0.49)	4.84 (0.52)	0.94	5.02 (0.54)	4.91 (0.54)	5.08 (0.53)	0.96
Self-leadership	4.70 (0.64)	4.62 (0.61)	4.78 (0.65)	0.81	4.96 (0.61)	4.88 (0.59)	5.01 (0.61)	0.83
Caring disposition	4.94 (0.56)	4.84 (0.55)	5.01 (0.55)	0.90	5.15 (0.57)	5.06 (0.61)	5.20 (0.54)	0.93
Character strength	4.67 (0.53)	4.59 (0.53)	4.73 (0.52)	0.88	4.94 (0.57)	4.80 (0.57)	5.02 (0.56)	0.91
Beliefs and values of service leadership	4.96 (0.61)	4.94 (0.60)	4.98 (0.63)	0.89	5.23 (0.65)	5.15 (0.69)	5.29 (0.62)	0.94

Note: Overall = overall sample; FTF = face-to-face; nFTF = non-face-to-face.

**Table 3:** Descriptive information of subjective outcome evaluation variables.

Dependent Variables	Mean (SD)			α
	Overall	FTF	nFTF	Overall
Perceptions of subject content	4.26 (0.61)	4.20 (0.59)	4.30 (0.61)	0.94
Perceptions of Teachers’ performance	4.51 (0.58)	4.52 (0.55)	4.51 (0.60)	0.97
Perceptions of Subject effectiveness	4.23 (0.59)	4.18 (0.60)	4.26 (0.58)	0.97
Overall satisfaction	4.02 (0.81)	3.90 (0.89)	4.08 (0.76)	-
Willing to recommend to others	3.75 (0.89)	3.57 (0.93)	3.85 (0.85)	-
Willing to take a similar course	4.19 (0.67)	4.16 (0.66)	4.21 (0.68)	-

Note: Overall = overall sample; FTF = face-to-face; nFTF = non-face-to-face.

**Table 4:** Results of repeated measure analyses on objective outcome evaluation.

Dependent Variables	Change		Service Mode		Change X Service Mode	
	F _(df)_	η_p_^2^	F _(df)_	η_p_^2^	F _(df)_	η_p_^2^
PYD (overall level)	87.46 *** ^(1214)^	0.29	7.07 ** ^(1214)^	0.03	1.98 ^(1214)^	0.01
Social competence	38.13 *** ^(1214)^	0.15	7.94 ** ^(1214)^	0.04	5.42 ^(1214)^	0.03
Emotional competence	49.28 *** ^(1214)^	0.19	8.56 ** ^(1214)^	0.04	1.31 ^(1214)^	0.01
Cognitive competence	64.21 *** ^(1214)^	0.23	5.48 ** ^(1214)^	0.02	2.32 ^(1214)^	0.01
Behavioral competence	52.28 *** ^(1214)^	0.20	8.99 ** ^(1214)^	0.02	1.36 ^(1214)^	0.01
Moral competence	10.60 ** ^(1214)^	0.05	1.41 ^(1214)^	0.01	3.34 ^(1214)^	0.02
Self-determination	48.52 *** ^(1214)^	0.19	10.52 ** ^(1214)^	0.05	1.14 ^(1214)^	0.01
Clear and positive identity	69.42 *** ^(1214)^	0.25	3.62 ^(1214)^	0.01	5.19* ^(1214)^	0.02
Belief in the future	23.53 *** ^(1214)^	0.10	4.81* ^(1214)^	0.02	1.74 ^(1214)^	0.01
Spirituality	15.77 *** ^(1212)^	0.07	0.00 ^(1212)^	0.00	0.14 ^(1212)^	0.00
Resilience	27.02 *** ^(1211)^	0.12	2.80 ^(1211)^	0.01	0.24 ^(1211)^	0.00
Life satisfaction	46.36 *** ^(1214)^	0.18	1.66 ^(1214)^	0.01	0.00 ^(1214)^	0.00
Service leadership (overall level)	72.64 *** ^(1211)^	0.26	3.17 ^(1211)^	0.01	0.03 ^(1211)^	0.00
Self-leadership	57.72 *** ^(1211)^	0.20	6.04 * ^(1211)^	0.03	0.19 ^(1211)^	0.00
Caring disposition	38.65 *** ^(1211)^	0.16	7.09 ** ^(1211)^	0.03	0.22 ^(1211)^	0.00
Character strength	69.64 *** ^(1210)^	0.25	6.55 * ^(1210)^	0.03	0.74 ^(1210)^	0.00
Service leadership beliefs	41.47 *** ^(1209)^	0.17	1.44 ^(1209)^	0.01	0.01 ^(1209)^	0.00

Note: Change = pretest-posttest change; Service mode: 0 = FTF; 1 = nFTF. * *p* < 0.05; ** *p* < 0.01; *** *p* < 0.05.

**Table 5:** The number of students who provided positive responses.

Perceptions of Course Qualities (PC)	Positive Responses (n [%])	
	Face-to-Face	Non Face-to-Face
The objectives of the curriculum are very clear.	117 (93.6%)	203 (93.1%)
The design of the curriculum is very good.	105 (84.0%)	196 (90.3%)
The activities were carefully arranged.	105 (84.0%)	193 (87.7%)
The classroom atmosphere was very pleasant.	108 (86.4%)	201 (92.2%)
There was much peer interaction amongst the students.	103 (82.4%)	183 (84.3%)
I participated actively during lessons (including discussions, sharing, games, etc.).	110 (88.0%)	193 (88.1%)
I was encouraged to do my best.	107 (87.0%)	204 (92.7%)
The learning experience I encountered enhanced my interest towards the lessons.	104 (83.2%)	198 (90.4%)
Overall speaking, I have very positive evaluation of the program.	111 (89.5%)	200 (92.2%)
On the whole, I like this curriculum very much.	103 (82.4%)	193 (88.5%)
**Perception of Teachers’ Qualities (PT)**	**Face-to-Face**	**Non Face-to-Face**
The lecturer(s) had a good mastery of the curriculum.	116 (92.8%)	208 (94.5%)
The lecturer(s) was (were) well prepared for the lessons.	117 (93.6%)	207 (94.1%)
The teaching skills of the lecturer(s) were good.	116 (92.8%)	207 (94.5%)
The lecturer(s) showed good professional attitudes.	115 (92.7%)	207 (95.0%)
The lecturer(s) was (were) very involved.	119 (95.2%)	209 (95.4%)
The lecturer(s) encouraged students to participate in the activities	118 (94.4%)	209 (95.4%)
The lecturer(s) cared for the students.	117 (93.6%)	205 (93.6%)
The lecturer(s) was (were) ready to offer help to students when needed.	119 (95.2%)	210 (96.3%)
The lecturer(s) had much interaction with the students.	119 (96.0%)	203 (92.7%)
Overall speaking, I have very positive evaluation of the lecturer(s).	117 (93.6%)	209 (95.0%)
**Perceptions of the Effectiveness (PE)**	**Face-to-Face**	**Non Face-to-Face**
It has enhanced my social competence.	115 (92.0%)	204 (93.2%)
It has improved my ability in expressing and handling my emotions.	109 (87.2%)	195 (88.6%)
It has enhanced my critical thinking.	103 (82.4%)	192 (87.7%)
It has increased my competence in making sensible and wise choices.	109 (87.9%)	197 (90.0%)
It has helped me to make ethical decision.	108 (86.4%)	202 (91.8%)
It has strengthened my resilience in adverse conditions.	110 (88.0%)	197 (89.5%)
It has strengthened my self-confidence.	110 (88.0%)	192 (87.7%)
It has helped me to face the future with a positive attitude.	109 (87.2%)	190 (86.8%)
It has enhanced my love for life.	98 (78.4%)	173 (79.4%)
It has helped me explore the meaning of life.	93 (74.4%)	174 (79.1%)
It has enhanced my ability of self-leadership.	110 (88.7%)	203 (92.7%)
It has helped me cultivate compassion and care for others.	114 (91.2%)	208 (94.5%)
It has helped me enhance my character strengths comprehensively.	110 (83.0%)	198 (90.4%)
It has enabled me to understand the importance of situational task competencies, character strength and caring disposition in successful leadership.	114 (91.2%)	205 (94.0%)
It has promoted my sense of responsibility in serving the society.	105 (85.4%)	205 (93.6%)
It has promoted my overall development.	114 (92.7%)	202 (92.7%)
The theories, research and concepts covered in the course have enabled me to understand the characteristics of successful leader.	112 (91.8%)	208 (95.0%)
The theories, research and concepts covered in the course synthesize the characteristics of successful leader.	114 (91.2%)	202 (93.1%)
**Overall satisfaction**	**Face-to-Face**	**Non Face-to-Face**
On the whole, are you satisfied with this course?	110 (88.7%)	194 (88.2%)
**Willingness**	**Face-to-Face**	**Non Face-to-Face**
Will you suggest your friends to take this course?	94 (75.8%)	180 (81.8%)
Will you participate in similar courses again in the future?	73 (58.9%)	160 (72.7%)

Notes: For the perceptions of subject content, teachers’ performance, and subject effectiveness, the positive response includes the options of 4 (agree) and 5 (strongly agree); for overall satisfaction, the positive response includes options of 4 (satisfied) and 5 (very satisfied); for willingness, for overall satisfaction, the positive response includes options of 4 (will) and 5 (definitely will).

## Data Availability

The data presented in this study are available on request from the corresponding author. The data are not publicly available due to privacy.

## Supplementary Materials

The following are available online at https://www.mdpi.com/1660-4601/18/4/2114/s1, Table S1: Learning outcomes of the subject, Table S2: Sample items of objective outcome evaluation form.
