# Supplementary material for: Serving Children and Adolescents in Need during the COVID-19 Pandemic: Evaluation of Service-Learning Subjects with and without Face-to-Face Interaction
Source: Int J Environ Res Public Health. 2021 Feb 22;18(4):2114. doi: 10.3390/ijerph18042114 (PMC7926360; doi:10.3390/ijerph18042114)
Supplement: Supplementary file 1 [file ijerph-18-02114-s001.pdf]

Supplementary Material

Table S1. Learning outcomes of the subject

Upon completion of the subject, students will be able to:
1) address the needs of the service recipients through service delivery;
2) link the service experiences with academic course materials;
3) appreciate and respect people from diverse background;
4) integrate academic learning (e.g., knowledge on service leadership) into the service experience and activities;
5) demonstrate empathy and care by providing community service;
6) apply the skills (e.g., active listening) and knowledge (e.g., moral character and competencies) they have acquired in university education to deal with complex issues in the service setting;
7) reflect on their service leadership qualities through service learning;
8) work effectively with different parties (e.g., students, teachers, families and community partners) when preparing and delivering service.

Table S2. Sample items of objective outcome evaluation form

Variable	Sample item (1 item)
Social competence	我懂得怎樣與人溝通。<br>I know how to communicate with others.
Emotional competence	當我不開心時，我能夠適當地表達我的情緒。<br>When I am unhappy, I can appropriately express my emotions.
Cognitive competence	我懂得從不同角度去看事物。<br>I know how to see things from different angles.
Behavioral competence	我能夠以開放的態度面對批評。<br>I can face criticisms with an open mind.
Moral competence	對於自己的行為，我有較高的道德要求。<br>I have high moral standards about my behaviors.
Self-determination	我有能力作出明智的抉擇。<br>I am capable to make wise choices.
Clear and positive identity	我是一個有自信的人。<br>I am a person with self-confidence.
Belief in the future	我有信心解決我將來會面對的問題。<br>I have confidence to solve my future problems.
Spirituality	我已經找到人生的目標。<br>I have found my purpose in life.
Resilience	面對困難時，我不會輕易放棄。<br>When I face difficulties, I do not give up easily.
Self-leadership	我明白自我發展的重要性。<br>I understand the importance of self-development.
Caring disposition	我容易察覺到別人的需求。<br>I am sensitive to others' needs.
Character strength	我樂於學習新知識。<br>I open my mind to embrace new knowledge.
Service Leadership beliefs	領導是一種對自我、他人、團體以及社會的服務。<br>Leadership is a service for self, others, groups and the society.
Life satisfaction	我的生活狀況是極好的。<br>The conditions of my life are excellent.
